# Molecular epidemiology of antibiotic-resistant genes and potent inhibitors against TEM, CTX-M-14, CTX-M-15, and SHV-1 proteins of *Escherichia coli* in district Peshawar, Pakistan

**DOI:** 10.1016/j.sjbs.2021.07.028

**Published:** 2021-07-14

**Authors:** Noor Rehman, Sadiq Azam, Amjad Ali, Ibrar khan, Muhammad Asghar, Momena Ali, Muhammad Waqas, Farman Ullah, Gul e Sehra

**Affiliations:** a Centre of Biotechnology and Microbiology, University of Peshawar, Pakistan; b Department of Biotechnology & Genetic Engineering, Hazara University, Mansehra, Pakistan; c Department of Genetics, Hazara University Mansehra, Pakistan; d Department of Pathology, Khyber Teaching Hospital, Peshawar, Pakistan; e Department of Pharmacy, Kohat University of Science and Technology, Kohat, Pakistan

**Keywords:** *E. coli*, Antibiotic-resistant genes, Drug designing, RECAP synthesis, Docking simulation, Potent inhibitors

## Abstract

The Extended Spectrum Beta-Lactamases (ESBLs) producing bacteria is an issue of concern for clinicians resulting in minimize the treatment options. To overcome resistance mechanisms, novel inhibitors with good Absorption, Distribution, Metabolism, Excretion, and Toxicity (ADMET) properties must inhibit the ESBLs resistant genes. The current study aimed to identify the antibiotic resistance genes of ESBLs producing *E. coli* and a single inhibitor was designed to inhibit all the resistant proteins. The results showed that 42.9% ESBL producers had CTX-M (69.9%), TEM (63.4%), SHV (34.5%) and CTX-M-14 (17.5%) genes. The ESBLs producing isolates were resistant to cephalosporins, quinolones, and sulfonamide with Minimum Inhibitory Concentration (MICs) ranging from 64 to >256 μg/ml. To design multi inhibitory ligands, RECAP synthesis was used for the de-novo discovery of 1000 inhibitors database. Protein crystal structures were retrieved from Protein Data Base (PDB). Lipinski’s rules of five were applied to the novel inhibitors database to improve the ADMET properties. The novel inhibitors database was selected for docking simulations. Placement of the ligand was used by the London dG algorithm implemented in Molecular Operating Environment (MOE), while GBVI/WSA dG algorithm was used for final refinement. Based on docking score, visual inspection of ligands interaction with key residues, binding affinity, and binding energy of ligands with proteins, ten compounds were selected for ESBLs proteins with best ADMET properties, binding energy, and binding affinity the reported ones. These hits compounds have unique scaffolds and are predicted to be a starting point for developing potent inhibitors against antibiotic-resistant proteins.

## 1 Introduction

*Escherichia coli* are Gram-negative bacilli belong to Enterobacteriaceae, commonly present in the human gut. It comprises nonpathogenic strains that form part of the normal flora, but some pathogenic strains can cause life-threatening infections in humans. The most common diseases caused by *E. coli* are; Urinary Tract Infections (UTIs) (Foxman, 2010), enteric infections, bacteremia, nosocomial pneumonia, cholecystitis, cholangitis, peritonitis, cellulitis, osteomyelitis, infectious arthritis, and neonatal meningitis (Kim, 2012). These infections are commonly treated with antibiotics, but improper use of these antibiotics has developed resistance in *E. coli* strains resulting in severe health issues worldwide (El Kholy et al., 2003). The prevalence and antibiogram of *E. coli* reveal massive global variations in different populations (Erb et al., 2007). A high rate of antibiotics resistance has been observed in Pakistan against CIP (49%), cefpodoxime (38%), CTX (36%), and ceftriaxone (34%) (Shah, 2002).

The *β*-lactams antibiotics are widely used in health care units for their efficacy against various Gram-negative bacteria. Unluckily, *β*-lactam drugs have developed multidrug resistance due to the production of *β*-lactamases like ESBLs (De Kraker et al., 2010). The ESBLs are a group of *β*-lactamases produced by Gram-negative bacteria, i.e., capable of hydrolyzing penicillin, aztreonam, and expanded spectrum cephalosporins (Hsueh et al., 2011). Moreover, these enzymes are not inhibited by other antibiotics like amino-glycosides, sulfonamide, and quinolones (Lautenbach et al., 2001).

The prevalence of ESBLs producing *E. coli* (ESBLs-Ec) is increasing rapidly globally (Kluytmans et al., 2012), ranging from 30 to 36% among clinical isolates in Pakistan (Jabeen et al., 2005, Pitout and Laupland, 2008). A high prevalence of 35.8% was reported in Saudi Arabia (Hassan and Abdalhamid, 2014). The prevalence rates of ESBLs-Ec are different in different geographic areas starting from low rates of 3–8% in Sweden, Japan, and Singapore to much higher rates of 30-60% in America, 34% in Portugal, and 58% in Turkey (Paterson and Bonomo, 2005).

These ESBLs genes are carried either on a chromosome or acquired via plasmid (Tamang et al., 2011). The Gram-negative bacteria are resistant to β-lactam drugs due to the production of CTX, SHV, and TEM β-lactamases encoded by CTX, SHV, and TEM genes, respectively. The CTX-M gene is the most widespread type of ESBLs in humans (Michael et al., 2015, Shaikh et al., 2015).

A high frequency (71%) of the CTX-M gene was reported in Saudi Arabia (Bindayna et al., 2010). Another study also observed the high incidence of CTX-M gene (59.32%) while both CTX-M and SHV genes were detected in 20.43% isolates of *E. coli* (Kaur and Aggarwal, 2013). A high prevalence of CTX-M, TEM and SHV genes was also observed in Lahore, Pakistan (Abrar et al., 2019). In Pakistan, the high frequency of ESBLs-related infections has been reported in the last 20 years (Abrar et al., 2017).

After completing molecular characterization, a variety of new potent drugs have been discovered to overcome antibiotic resistance of ESBLs-Ec. In the drug discovery process, combinatorial chemistry is a well-established strategy for generating new lead molecules. Retrosynthetic Combinatorial Analysis Procedure (RECAP) Analysis and Synthesis is a new computational tool used to create fragments molecules and recombine these molecules in various ways to synthesize reasonable novel chemical structures (Lewell et al., 1998). It can give compounds with high hit rates during screening libraries of in-silico compounds compared to traditional large-scale screening (Shekhar, 2008). Thus, with the help of available computational tools, antibiotic resistance can be efficiently handled and will support clinicians to overcome the resistance mechanisms.

Therefore, the current study was designed to determine the antimicrobial susceptibility patterns of ESBLs producing E. coli and search for novel inhibitors, as it is necessary regularly for adequate clinical treatment and management.

## 2 Materials and methods

### 2.1 Samples collection and inoculation

A total of 3278 clinical samples were collected from blood, Cerebrospinal Fluid (CSF), urine, pus, sputum, and infected wounds and transported to the microbiology laboratory of Khyber Teaching Hospital Peshawar for further processing. Patients undergoing antibiotic therapy or suffering from enteric infections were excluded from the study. The collected samples were inoculated on Cysteine Lactose Electrolyte-Deficient medium (CLED), MacConkey agar, blood agar and incubated at 37 °C for 24hrs (Cheesbrough, 2006). Specimens like CSF, pus, wound, and respiratory specimens like sputum were inoculated on blood agar and MacConkey agar plates and incubated at 37 °C for 24hrs. Samples like blood, CSF, and other body fluids were inoculated in a culture medium of automated blood culture system (Versa Trek, USA). The inoculated bottles were subjected to incubation at 37 °C for five days and observed for gas production. Pure isolated colonies were analyzed for their morphology and further subjected to Gram staining. Later on, these bacterial cultures were subjected to different biochemical tests using Analytical Profile Index (API) 10S strips for confirmation (Alnahass et al., 2016). Moreover, these phenotypically identified isolates were confirmed by PCR using specific primers for the *uidA* gene.

### 2.2 Phenotypic screening for ESBLs production

In phenotypic detection, the isolates resistant to CAZ or CTX were subjected to E-test for ESBLs production. The test organism was inoculated on Mueller-Hinton Agar (MHA) plate. The E-test strips were placed on the plates and incubated at 37 °C for 24hrs. A ratio of the MICs of the cefotaxime/inhibitor (CT/CTL), ceftazidime/inhibitor (TZ/TZL), and cefepime (PM/PML) of ≥8 or the presence of a phantom zone was interpreted as positive for ESBLs production (Cormican et al., 1996).

### 2.3 Antimicrobial susceptibility testing

All isolates were subjected to antimicrobial-susceptibility testing against selected antibiotics by Kirby Bauer disc diffusion technique. The bacterial suspension was inoculated on MHA plates and the antibiotic discs were placed on plates at equal distances. The results were interpreted after overnight incubation at 37˚C by measuring the inhibition zone diameter as per Clinical and Laboratory Standard Institute (CLSI) 2019 guidelines (Cusack et al., 2019).

### 2.4 Determination of minimum inhibitory concentrations

The MICs test strips were used for the determination of MICs of selected antibiotics. The strips containing an exponential gradient of antimicrobial agents were placed on the surface of an inoculated MHA plate. These plates were incubated for 24hrs at 37 °C, and the MICs were recorded immediately from the scale (μg/mL) at the point where the edge of the inhibition ellipse intersects the MIC strip (Yan et al., 2002).

### 2.5 Molecular identification of E. coli

All the isolates were sub-cultured on blood agar and incubated overnight at 37˚C. The genomic DNA was extracted using a GeneJet genomic DNA purification kit (Thermo Scientific, USA). The DNA integrity was determined by gel electrophoresis in 1% agarose (w/v), and purified DNA was stored at −20 °C (Bashir et al., 2012, Chen and Griffiths, 1998). The *E. coli* isolates were confirmed by amplifying the *β*-D-glucouronidase (*uidA*) gene using specific primers. The final PCR reaction was done in 27 µl final reaction volume containing; 12.5 µl Taq master mix (Bioron, Germany), 0.5 µl of forward and reverse primers, 2 µl of DNA template and PCR grade water. Amplification was carried out using specific primer sequences under the optimized conditions (Bashir et al., 2012) (Table 1).

**Table 1:** Primers used for confirmation and molecular characterization of ESBLs-Ec.

Gene	Gene Primers (5′→3′)	Product Size	Annealing
*uidA*	F: ATCACCGTGGTGACGCATGTCGC R: CACCACGATGCCATGTTCATCTGC	486	54 °C
*Bla-SHV*	F: CACCACGATGCCATGTTCATCTGC R: TCGCCTGTGTATTATCTCCC	768	52 °C
*Bla-TEM*	F: CGCAGATAAATCACCACAATG R: GTCTATTTCGTTCATCCATA	247	54 °C
*Bla*-CTX-M	F: ATGTGCAGCACCAGTAAAGT R: ACCGCGATATCGTTGGTGG	545	54 °C
*Bla*-CTX-M-14	F: CTGATGTAACACGGATTGACC R: CGATTTATTCAACAAAACCAG	871	58 °C

### 2.6 Molecular characterization and sequencing of ESBLs gene (s)

Molecular identification of ESBLs genes was carried out by PCR, using the specific primers (Table 1). All the isolates were screened for the selected ESBLs resistance genes; SHV, TEM, CTX-M, and CTX-M-14 (Kozak et al., 2009, Leflon-Guibout et al., 2004, Mendonça et al., 2007, Van et al., 2008).

### 2.7 Gel electrophoresis

The amplified products were electrophoresed at 110 V for 40–60 min on 1.5% agarose suspended in TAE buffer (1X). Gels were stained with ethidium bromide solution, and bands were visualized and photographed by gel documentation system (Bio-Rad Milan, Italy) using GeneSnap software. The amplicon sizes were determined by matching with DNA ladder (100 bp) (Gangoue-Pieboji et al., 2006).

### 2.8 Designing of potent inhibitors for ESBL proteins

For drug designing, crystal structures were retrieved from PDB (https://www.rcsb.org). TEM PDB-ID 1ERM (Ness et al., 2000), CTX-M-14 PDB-ID 4XXR (Lewandowski et al., 2015), CTX-M-15 PDB-ID 4HBU (Lahiri et al., 2013), and SHV-1 PDB-ID 2ZD8 with the resolution of 1.05Å (Nukaga et al., 2008). The crystal structure of the *E. coli* β-lactamase SHV-1 gene is not yet reported; that is why the *K. pneumoniae β*-lactamase SHV-1 protein crystal structure was selected.

### 2.9 Data analysis

To visualize the crystal structures MOE 2019-0102, PyMOL software, Maestro from Schrodinger suite, was used (Bello, 2018, DeLano, 2002).

### 2.10 Electrostatic maps

This application works on the principle of nonlinear Poisson-Boltzmann Equation (Eq. (1)). For predicting electrostatically preferred locations of H-bond acceptor, H-bond donor and hydrophobic atoms in the active site, electrostatic Maps were generated for each protein using 4.5 Å receptor grids MOE2019.0102. Before calculation, the system was partially charged (qi). Furthermore, this equation yields electrostatic potential (u) and then use to produce predictive maps as under.

#### 2.10.1 Acceptor map

An isocontour of the potential (qO u + vO), in which vO indicates van der Waals potential while qO indicates the partial charge of the oxygen ion.

#### 2.10.2 Donor map

An isocontour of the potential (qH u + vH), in which vH indicates van der Waals potential and qH is the partial charge of the hydrogen ion and vH is the van der Waals potential of the hydrogen atom.

#### 2.10.3 Hydrophobic map

An isocontour of the potential – (qO + qH) u + vC in which vC indicates van der Waals potential of a carbon atom.

(1) $$\nabla .\varepsilon^{\nabla }\emptyset +\sum_{i}q_{i}C_{i}e^{-\frac{q_{i}\emptyset +u_{i}}{\mathrm{kT}}}+p=0$$

This application computes suitable positions like positive, neutral, and negative charges in the ligand and binding site of protein using Eq. (1). The hot spots present in the protein pocket indicate binding. In hot spots, red indicates Acceptor/Negative, blue for Donor/Positive, and green for Neutral/Hydrophobic. The protein crystal structures were recovered from the RCSB PDB server having ligand bind in it were selected for the electrostatic map to find the binding residues pattern in the active pocket.

### 2.11 Retrosynthetic combinatorial analysis procedure (RECAP) analysis and synthesis

To generate novel inhibitors database, 21 reported β-lactamases inhibitors were recovered from the Pubchem server (https://pubchem.ncbi.nlm.nih.gov/) with unique PubChem CID and were kept in the inhouse database in mdb format. RECAP Analysis and Synthesis implemented in MOE 2019 were applied on the above-reported inhibitors as part of a de novo discovery methodology. One thousand novel structures were generated and were saved in mdb format. All the structures in the database were energy minimized, and the hydrogens were added to it using the MOE2019.0102.

### 2.12 ADMET properties

To select inhibitors having good ADMET properties, Lipinski’s rules of five were applied using the MOE 2019 descriptor calculator, on the output database generated from the RECAP synthesis (Lipinski, 2004).

### 2.13 Docking validation and simulations

Before docking the novel ligands in the proteins active site, the docking protocol of MOE2019.0102 was validated through redocking, the reported inhibitors in the crystal structures. The method of Rigid body pose generation was used for Protein-ligand docking. The validation of docking was reported in RMSD of the re-docked and reported ligand superimposition. The retrieved output database from RECAP analysis and synthesis was docked in the active sites of each protein after the docking validation. To generate tens of thousands of ligand poses around the restraints to restrict the binding site region, the tool of Fast Fourier Transforms (FFT) was selected. The (rigid body) top 10 poses were refined with R-Field electrostatics, and the final Refined (top 10 poses) were generated based on equation-2 GBVI/WSA dG solvation model as output. Where the transitional and rotational entropy gain/loss average is represented by “c”, the forcefields dependent constants are “α and β”, to calculate the currently loaded charges using εi = 1 constant dielectric, represented by coulombic electrostatic E_Coul_, GB/VI solvation model E_sol_ was used to calculate solvation electrostatic, E_vdW_ showed the van der Waals contributions and the surface area exposed was calculated by SA_weighted_.

(2) $$\Delta G\approx c+a\left[\frac{2}{3}\left(\Delta E_{\mathrm{coul}}+{\Delta E}_{\mathrm{sol}}\right)+\Delta E_{\mathrm{vdw}}+\beta \Delta SA_{\mathrm{weighted}}\right]$$

The docked compounds were saved in mdb format. Using the proxy triangle algorithm, 1 conformation was saved for each ligand using equation-3 London dG scoring methodology in MOE2019.0102 for refinement (Labute, 2008). Where rotational and translational entropy gain or loss is represented by “c”, ligand flexibility energy loss is E_flex_, hydrogen bonds geometric imperfections are f_HB_, ideal hydrogen bond energy is c_HB_, metal ligation imperfection is f_M_. In contrast, ideal metal-binding energy is c_M_, and the dissolution energy of i atom is D_i_.

(3) $$\Delta G=c+E_{\mathrm{flex}}+\sum_{h-bonds}c_{\mathrm{HB}}f_{\mathrm{HB}}+\sum_{m-lig}c_{M}f_{M}+\sum_{\mathrm{atomi}}\Delta D_{i}$$

The final ten compounds were selected based on the highest docking score, interaction with crucial residues visual inspection, binding energy, and binding affinity, potentially inhibiting all the MBLs proteins. These ten final hits have more tremendous binding energy, and binding affinity from the crystal structures reported inhibitors and can be used for experimental procedures.

### 2.14 Calculations of binding energy and binding affinity

The binding affinities were calculated using the equation-2 Generalized Born/Volume Integral (GB/VI) algorithm in MOE2019-0102 to determine the most potent ligand for each *β*-lactamases protein complex with ligands. All the non-bonded interaction energies are Generalized Born interaction between the protein residues, and the ligand molecules consist of coulomb electrostatic interaction. The binding energy and binding affinity were calculated and described in the unit (Kcal/Mol) for each hit after energy minimization (Wadood et al., 2017).

## 3 Results

### 3.1 Phenotypic results of ESBLs-Ec

The results of the prevalence analysis revealed that out of 3278 clinical samples, 573(17.5%) yielded the growth of *E. coli*. In a total of 573 isolates, 246 (42.9%) isolates were ESBLs positive. Out of 246 ESBLs-Ec isolates, 39.0% isolates were recovered from male patients while 61% from female. The highest rate of ESBLs production (29.3%) was observed among the age group 41–60 years, followed by the age group 21–40 years (27.2%). The majority of the ESBLs-Ec isolates were recovered from urine 151 (61.4%) followed by pus 72(29.3%). The results of the different clinical isolates of ESBLs-Ec revealed that 151(61.4%) patients had UTIs, 87(35.3%) patients had Severe Systemic Infections (SSIs), while 08(3.3%) patients had severe Pulmonary Infections (PIs). The majority of the ESBL-Ec isolates were detected in hospitalized patients 131(53.2%) as compared to OPD patients 115 (46.7%) (Table 2).

**Table 2:** Frequency distribution of ESBLs-Ec in different types of clinical isolates (n = 246).

Parameters	Frequency (n)	Percentage
Total *E. coli* isolates	573	17.5
**ESBL-Ec isolates**	246	42.9
**Gender**		
Male	96	39.0
Female	150	61.0
**Age Groups (years)**		
00 – 10	50	20.3
11 – 20	27	11.0
21 – 40	67	27.2
41 – 60	72	29.3
> 60	30	12.2
**Specimen Type**		
Pus	72	29.3
Urine	151	61.4
Wound	4	1.6
Sputum	8	3.3
Others	10	4.1
Fluids	1	0.4
**Clinical Features**		
UTI	151	61.3
SSIs	87	35.3
Pıs	8	3.3
**Patients Status**		
Indoor Patients	131	53.2
Outdoor Patients	115	46.7
**ESBLs Genes**		
CTX – M	172	69.9
TEM	156	63.4
SHV	85	34.5
CTX X-M – 14	43	17.5

### 3.2 Antimicrobial resistance in ESBLs-Ec isolates

The result of antibiogram revealed that ESBLs-Ec isolates were sensitive (100%) against TGC and MEM while resistant (100%) against antibiotics; FEP, AMP, CTX, ATM and CAZ. The high resistance was also observed in isolates tested against AMC (93.1%), SXT (93.1%), CIP (90.7%), LVX (86.6%), TOB (70.7%), DO (65.0%), CN (56.9%), C (51.6%), while some antibiotics; FOS (17.5%), SCF (26.8%), AK (37.3%) and TZP (45.4%) were effective against ESBLs-Ec isolates as shown in (Table 3).

**Table 3:** Antibiogram of ESBLs-Ec isolates against various antibiotics (n = 246).

Antibiotics	Symbols	Sensitivity		Resistance	
		Frequency	Percentage	Frequency	Percentage
Ampicillin	AMP	00	00	246	100
Amoxicillin-Clavulanate	AMC	17	6.9	229	93.1
Cefoperazone-Sulbactam	SCF	180	73.2	66	26.8
Piperacillin-Tazobactam	TZP	134	54.5	112	45.4
Cefepime	FEP	00	00	246	100
Cefotamixe	CTX	00	00	246	100
Ceftazidime	CAZ	00	00	246	100
Aztreonem	ATM	00	00	246	100
Meropenem	MEM	246	100	00	00
Gentamicin	CN	106	43.0	140	56.9
Tobramycin	TOB	72	29.3	174	70.7
Amikacin	AK	154	62.6	92	37.3
Doxycycline	DO	86	35.0	160	65.0
Ciprofloxacin	CIP	23	9.3	223	90.7
Levofloxacin	LEV	33	13.4	213	86.6
Cotrimoxazole	SXT	17	7.0	229	93.1
Chloramphenicol	C	119	48.4	127	51.6
Fosfomycin	FOS	203	82.5	43	17.5
Tigecycline	TGC	246	100	00	00

### 3.3 Determination of Minimum Inhibitory concentrations

The high MIC values were recorded for *β*-lactam drugs; CTX (MIC_50_ ≥ 128; MIC_90_ ≥ 256 µg/ml), CAZ (MIC_50_ ≥ 64; MIC_90_ ≥ 256 µg/ml) and non-*β*-lactam drugs; SXT (MIC_50_ ≥ 24; MIC_90_ ≥ 256 µg/ml), CIP (MIC_50_ ≥ 24; MIC_90_ ≥ 256 µg/ml), DO (MIC_50_ ≥ 16; MIC_90_ ≥ 192 µg/ml), CN (MIC_50_ ≥ 4; MIC_90_ ≥ 16 µg/ml) and AK (MIC_50_ ≥ 8; MIC_90_ ≥ 256 µg/ml) against ESBLs-Ec isolates. The low MIC values were recorded for MEM (MIC_50_ ≥ 0.125; MIC_90_ ≥ 0.75 µg/ml) and TGC (MIC_50_ ≥ 0.25; MIC_90_ ≥ 1.5 µg/ml). The MICs of cephalosporins for resistant isolates ranged from 64 to >256 μg/ml as shown in (Table 4).

**Table 4:** Minimum Inhibitory Concentration of antibiotics against ESBLs-Ec isolates (n = 246).

Antibiotics	Breakpoints			MIC_50_ (µg/ml)	MIC_90_ (µg/ml)	MIC Range (µg/ml)
	S	I	R			
CTX	≤ 1	2	≥4	128	256	4–256
CAZ	≤ 4	8	≥16	64	256	16–256
MEM	≤ 1	2	≥4	0.125	0.75	0.023–1
CN	≤ 4	8	≥16	4	16	0.064–140
AK	≤ 16	–	64	8	256	0.19–256
DO	≤ 4	8	≥16	16	192	0.125–256
CIP	≤0.25	0.5	≥1	24	256	0.25–256
SXT	≤ 2/38	–	≥4/76	24	256	0.19–256
TGC	≤ 2	–	–	0.25	1.5	0.023–2

### 3.4 Molecular characterization of ESBLs gene (s)

**Fig. 1:**
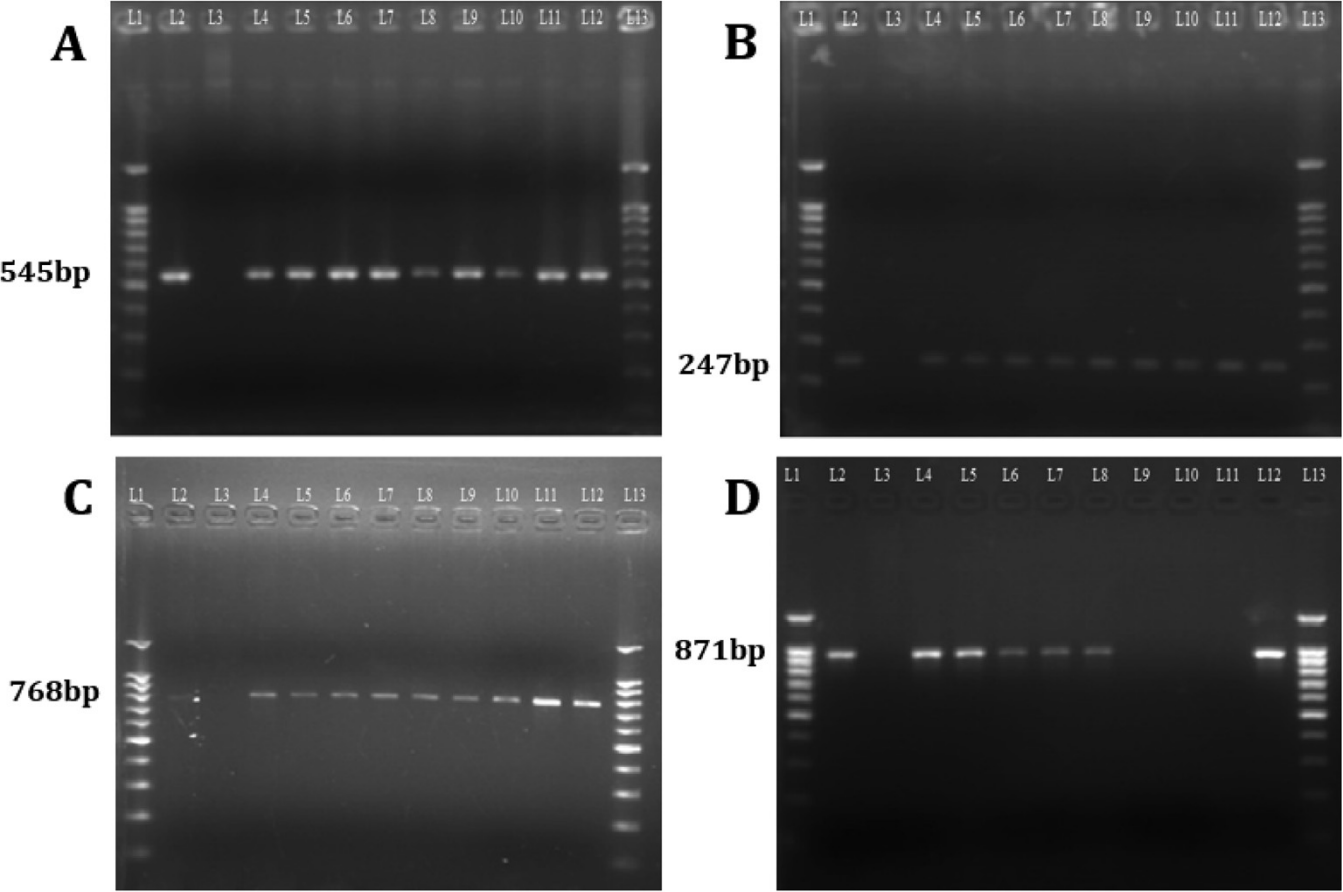
Gel electrophoresis of PCR products of the A) CTX-M, B) TEM, C) SHV, and D) CTX-M-14.

The targeted ESBLs genes were detected in 242 (98.37%) isolates out of 246. These isolates were positive for one or more genes, while 04 isolates were negative for targeted genes. The Bla-CTX-M was the most prevalent gene, observed in 172(69.9%), TEM in 156(63.4%), SHV in 85(34.5%), while CTX-M-14 was observed in 43(17.5%) isolates (Table 2 and Fig. 1). After sequencing, the reference sequences were aligned using the NCBI Blast; Bla-TEM gene with accession number MK878892, Bla-SHV gene with accession number MH460805, Bla-CTX-M gene with accession number MN200691, and Bla-CTX-M-14 gene with accession number NG_048929. The *E. coli* strain (ATCC 35218) was used as a positive control for the TEM gene, *K. pneumonia* strain (ATCC 700603) for SHV, CTX-M14, and CTX-M genes, while *E. coli* strain (ATCC 25922) was used as a negative control.

### 3.5 Designing of potent ESBLs inhibitors

**Fig. 2:**
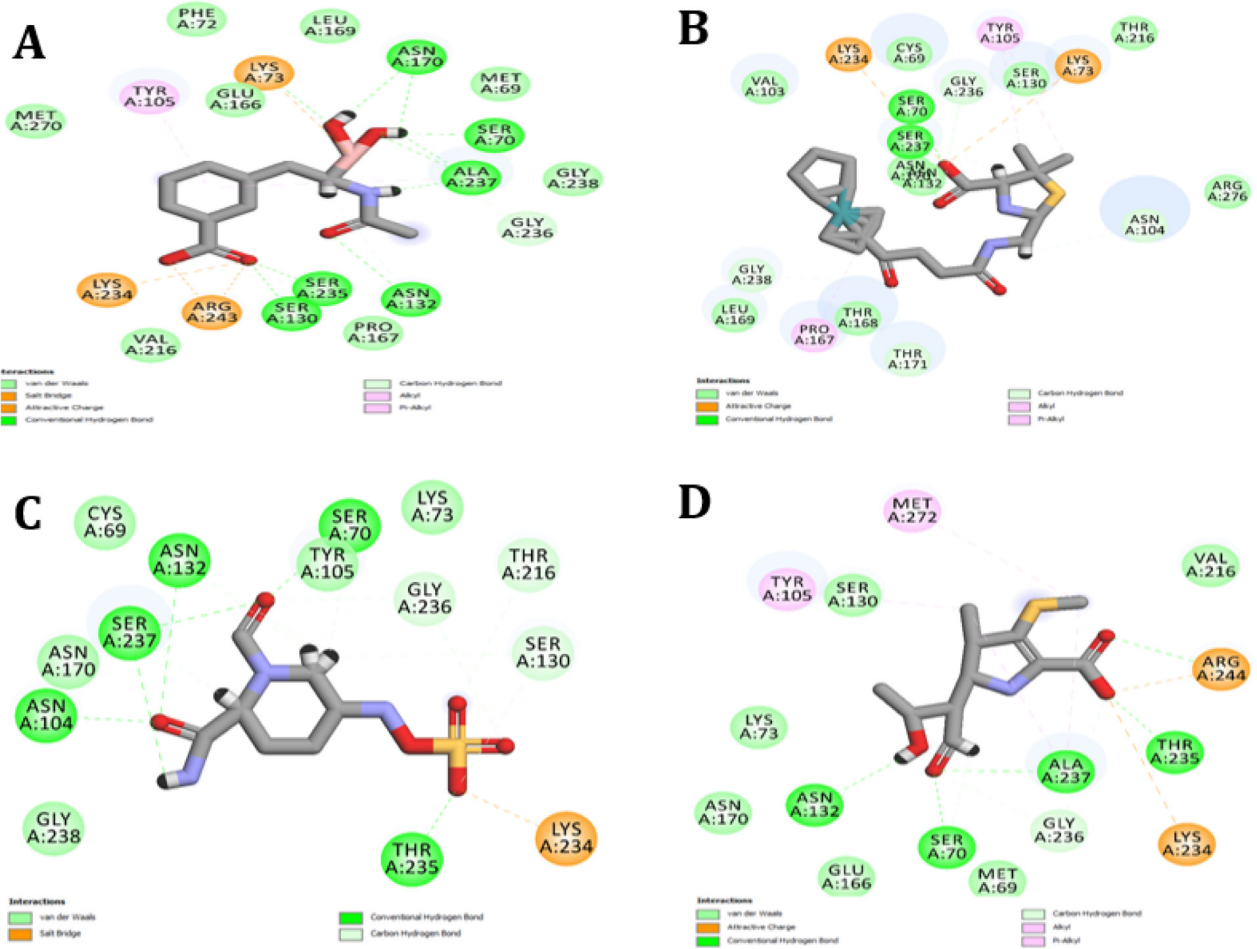
2D Interactions of the 1) TEM with Ethyl Boronic Acid 2) CTX-M-14 with ruthenium 3) CTX-M-15 with NXL and 4) SHV-1 Meropenem. The hydrogen bonds were shown within 3 Å distance in the interaction.

The resistance to antibiotics is an issue of concern and has involved interests for researchers worldwide. In the past, Insilco approaches have been used to predict strategies like targeting the protein crystal structures to design novel inhibitors to overcome the mechanism of resistance (Hassan Baig et al., 2016). To design multi inhibition small molecules for ESBLs proteins, crystal structures of the four selected proteins were retrieved from the RCSB PDB server. The reported ligand for the 1ERM Bla-TEM is Ethyl Boronic Acid, ruthenium (ligand) for 4XXR Bla-CTX-M-14, (2S, 5R)-1-formyl-5-[(sulfooxy) amino] piperidine-2-carboxamide (NXL) for 4HBU Bla-CTX-M-15, Meropenem for 2ZD8 Bla-SHV-1 is given in (Fig. 2).

### 3.6 Electrostatic maps

**Fig. 3:**
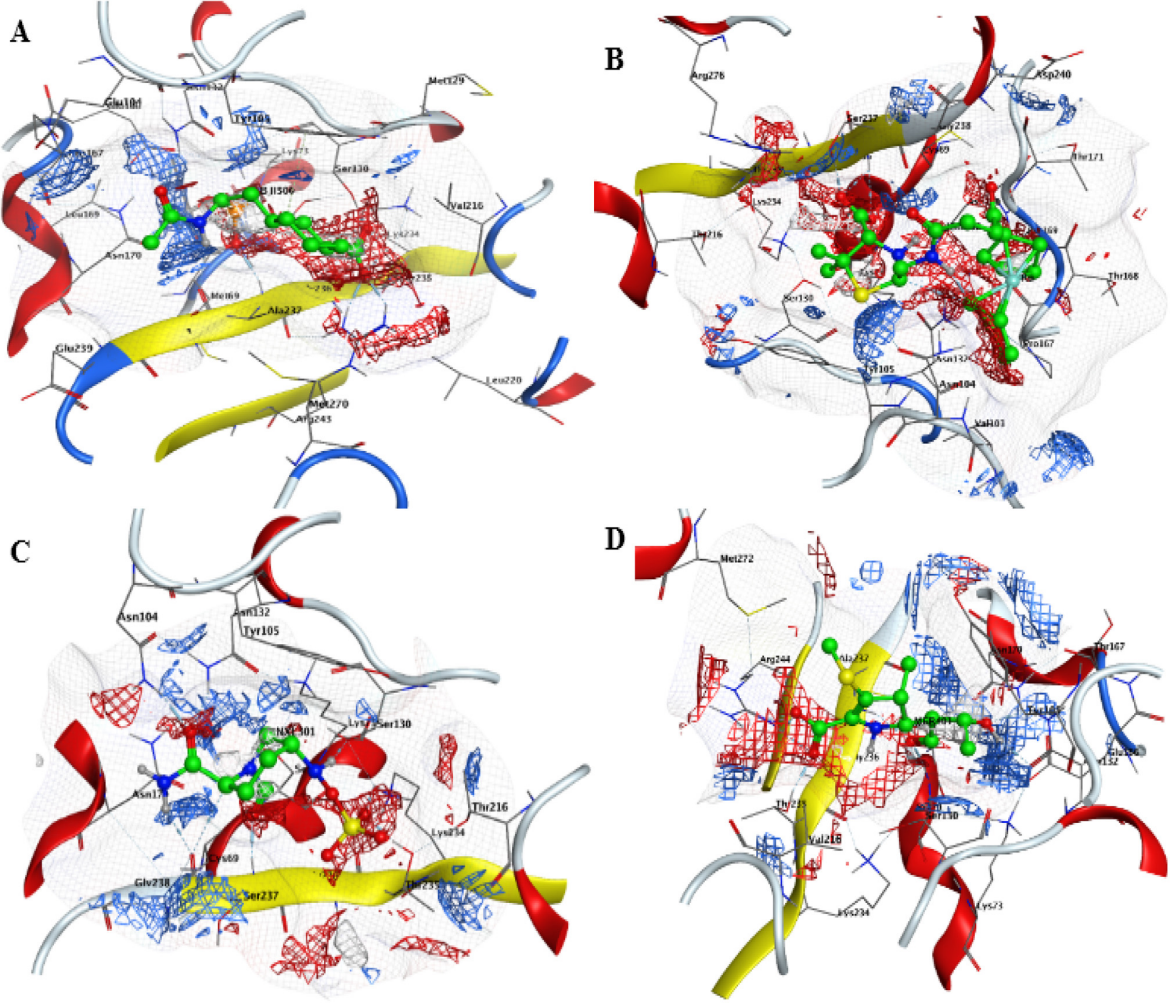
Electrostatic maps of A) TEM B) CTX-M-14C) CTX-M-15 and D) SHV-1 proteins. The electrostatic map was calculated for the active pocket of protein and ligand of beta-lactamase proteins having ligand in it. The color of hydrogen bond acceptor areas is red; the hydrogen bond donner atoms are blue, and the hydrophobic interaction atoms are represented by green color.

The electrostatic map was calculated for the active pocket of protein and ligand of all the selected beta-lactamase proteins (TEM, CTX-M-14, CTX-M-15, and SHV-1) having ligand in it (Fig. 3). The color of hydrogen bond acceptor areas is red, the hydrogen bond donor atoms are blue, and the hydrophobic interaction atoms are represented by green color.

### 3.7 Docking validation

**Fig. 4:**
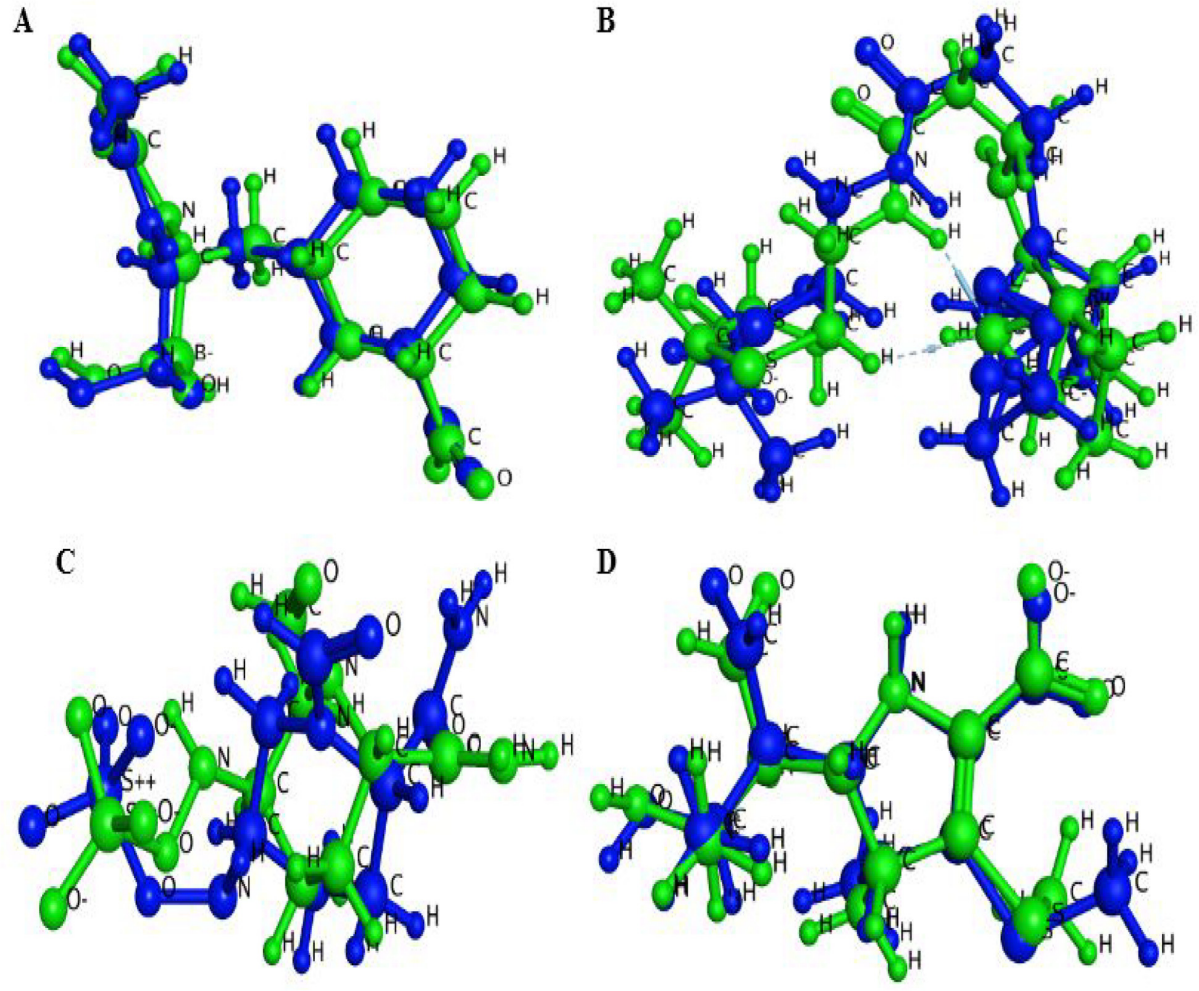
Docking validation of the A) Green TEM protein–ligand Ethyl Boronic Acid superposed with Blue redock B) Green CTX-M-14 protein–ligand ruthenium superposed with Blue redock C) Green CTX-M-15 protein–ligand NXL superposed with Blue redock D) Green SHV-1 protein–ligand Meropenem superposed with Blue redock.

The docking techniques were subjected for validation by docking the reported co-crystallized ligands of all targeted *β*-lactamases in the active pockets of each protein before docking (Fig. 4). The docked ligands of the proteins were in suitable range (0.3274 Å) for TEM, (0.5273 Å) for CTX-M-14, (0.8047 Å) for CTX-M-15 and (0.9144 Å) for Bla-SHV-1.

### 3.8 Docking simulation, prediction of binding energy and binding affinity

The residues interacting with the reported ligand in the crystal structure were selected as an active pocket of the proteins. The Novel compounds generated from RECAP synthesis were docked with the active pocket residues. The docked compound of 20% based on the lowest docking score was selected for visual inspection to identify the best interacting compounds. Based on docking score, binding energy, and binding affinity, the top 10 compounds were selected, which have promising interactions with selected proteins. To rank, the final compounds, binding energy, and binding affinity were calculated for each compound with the selected proteins shown in (Table 5).

**Table 5:** Binding energies, binding affinities and docking scores of the final 10 hits compounds.

Bla-TEM Portein					Bla-CTXM-14 Protein		
S. No.	Compound No	Docking Score	Binding Energy (Kcal/mol)	Binding Affinity (Kcal/mol)	Docking Score	Binding Energy (Kcal/mol)	Binding Affinity (Kcal/mol)
1	151	−7.71	−67.06	−8.82	−6.27	−69.70	−8.61
2	155	−7.95	−69.71	−9.02	−6.86	−62.18	−8.18
3	275	−7.97	−80.49	−9.48	−6.69	−71.42	−8.55
4	377	−7.87	−65.21	−8.58	−6.55	−51.61	−7.19
5	382	−7.99	−67.23	−8.80	−7.18	−61.04	−8.20
6	781	−7.05	−67.28	−8.75	−7.14	−70.74	−9.02
7	783	−8.03	−78.66	−9.43	−7.30	−69.95	−8.63
8	804	−7.85	−66.45	−8.61	−6.83	−67.74	−8.51
9	848	−8.08	−70.84	−8.98	−7.01	−72.54	−9.73
10	962	−7.35	−64.87	−8.62	−7.28	−73.81	−9.67
Ref			−56.72	−8.43		−50.69	−8.37

Bla- CTXM-15 Protein					Bla-SHV-1 Protein		

1	151	−7.27	−69.54	−9.30	−7.33	−61.29	−8.37
2	155	−6.92	−60.36	−8.04	−6.67	−66.10	−8.86
3	275	−7.19	−60.74	−8.30	−6.84	−91.98	−11.03
4	377	−6.73	−56.61	−8.01	−6.87	−52.03	−7.42
5	382	−6.99	−58.60	−7.69	−7.10	−66.18	−8.92
6	781	−7.28	−53.40	−8.21	−6.76	−64.77	−8.33
7	783	−8.21	−77.88	−9.79	−7.14	−78.19	−10.26
8	804	−7.75	−71.44	−9.21	−6.48	−71.48	−9.47
9	848	−6.93	−64.55	−8.32	−7.45	−61.87	−8.47
10	962	−7.51	−66.28	−8.79	−6.52	−66.44	−8.84
Ref			−46.89	−6.63		−50.05	−7.08

### 3.9 Docking score, binding energy, and binding affinity for TEM protein

**Fig. 5:**
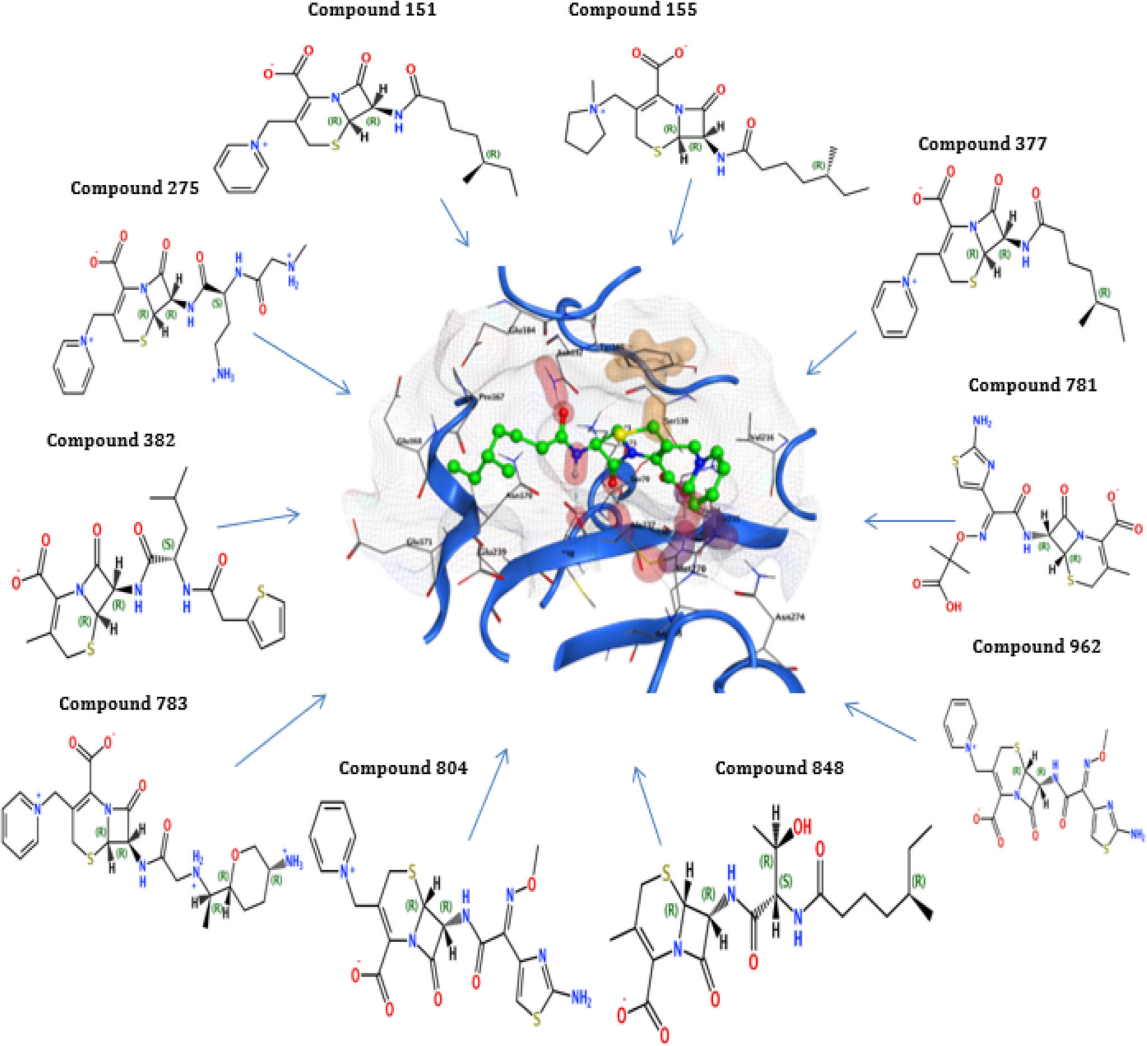
Interactions of the final retrieved hit compounds with target protein of Bla-TEM after docking. The final ten compounds were predicted as promising hits compounds with the best ADMET properties, binding energy, and binding affinity, showing best interactions with Bla-TEM protein. The final promising hits compounds blocked the resistance mechanism of Bla-TEM protein.

The highest binding energy (−80.49 kcal/mol) and binding affinity (−9.48 kcal/mol) were reported for TEM of compound 275, followed by compound 783 having binding energy (−78.66 kcal/mol), binding affinity (−9.43 kcal/mol), compound 848 with binding energy (−7084 kcal/mol), binding affinity (−8.98 kcal/mol). All the ten reported compounds showed the best binding energy and affinity (Table 5). The docking result of compound 848 for TEM protein revealed that the highest docking score of −8.08 was observed interacting with Ser130, Ser235, Tyr105, Ala237, and Arg243 of the active pocket having high binding energy (−70.84Kcal/mol) and affinity (−8.98Kcl/mol). The compound 783 for Bla-TEM also documented a high docking score (−8.03), binding energy (−78.66Kcal/mol), and affinity (−9.43Kcal/mol), which interact with Ser130, Ser235, Tyr105, Asn132, Gly236, Ala237, and Arg243 of the active pocket. The compounds 848 and 783 recorded the best interaction with critical residues of the TEM active site (Tables 5 and Fig. 5).

### 3.10 Docking score, binding energy, and binding affinity for CTX-M-14 protein

**Fig. 6:**
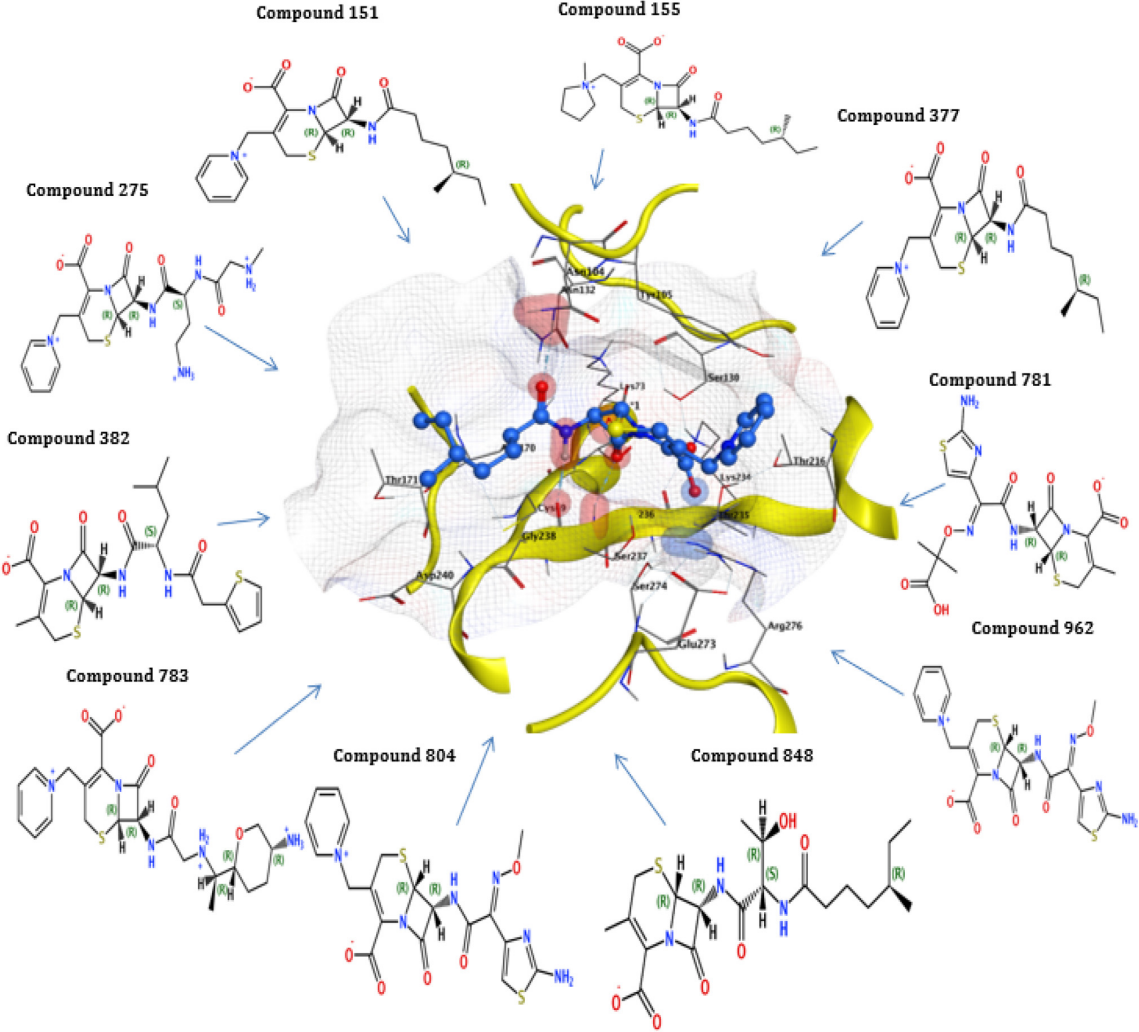
Interactions of the final retrieved hit compounds with target protein of Bla-CTX-M-14 after docking. The final ten compounds were predicted as promising hits compounds with the best ADMET properties, binding energy, and binding affinity, showing best interactions with Bla-CTX-M-14 protein. The final leading hits compounds blocked the resistance mechanism of Bla-CTX-M-14 protein.

The results of 4 compounds out of 10 selected compounds for bla-CTX-M-14 revealed that compound 962 has binding energy (−73.81 kcal/mol) with affinity (−9.67 kcal/mol), compound 848 has a binding energy of (−72.54 kcal/mol) with affinity (−9.73 kcal/mol), the compound 275 has a binding energy of (−71.42 kcal/mol) with affinity (−8.55 kcal/mol) and compound 781 has a binding energy of (−70.74 kcal/mol) with affinity (−9.02 kcal/mol). These four compounds have binding energy above than −70 kcal/mol (Table 5). The highest docking score (−7.30) of compound 783 was observed for Bla-CTX-M-14 with binding energy (−69.95Kcal/mol) and affinity (−8.63Kcal/mol) having interactions with Ser237, Tyr105, Arg276, and Arg243 of the active pocket. The second compound, 962 of Bla-CTX-M-14, documented docking score (−7.28), binding energy (−73.81Kcal/mol), and affinity (−9.67Kcl/mol) having interaction with Ser70, Ser237, Thr171, Asn104, and Arg276 of the active pocket. The compounds 783 and 962 showed good interaction with the essential residues of the CTX-M-14 active site (Tables 5 and Fig. 6).

### 3.11 Docking score, binding energy, and binding affinity for CTX-M-15 protein

**Fig. 7:**
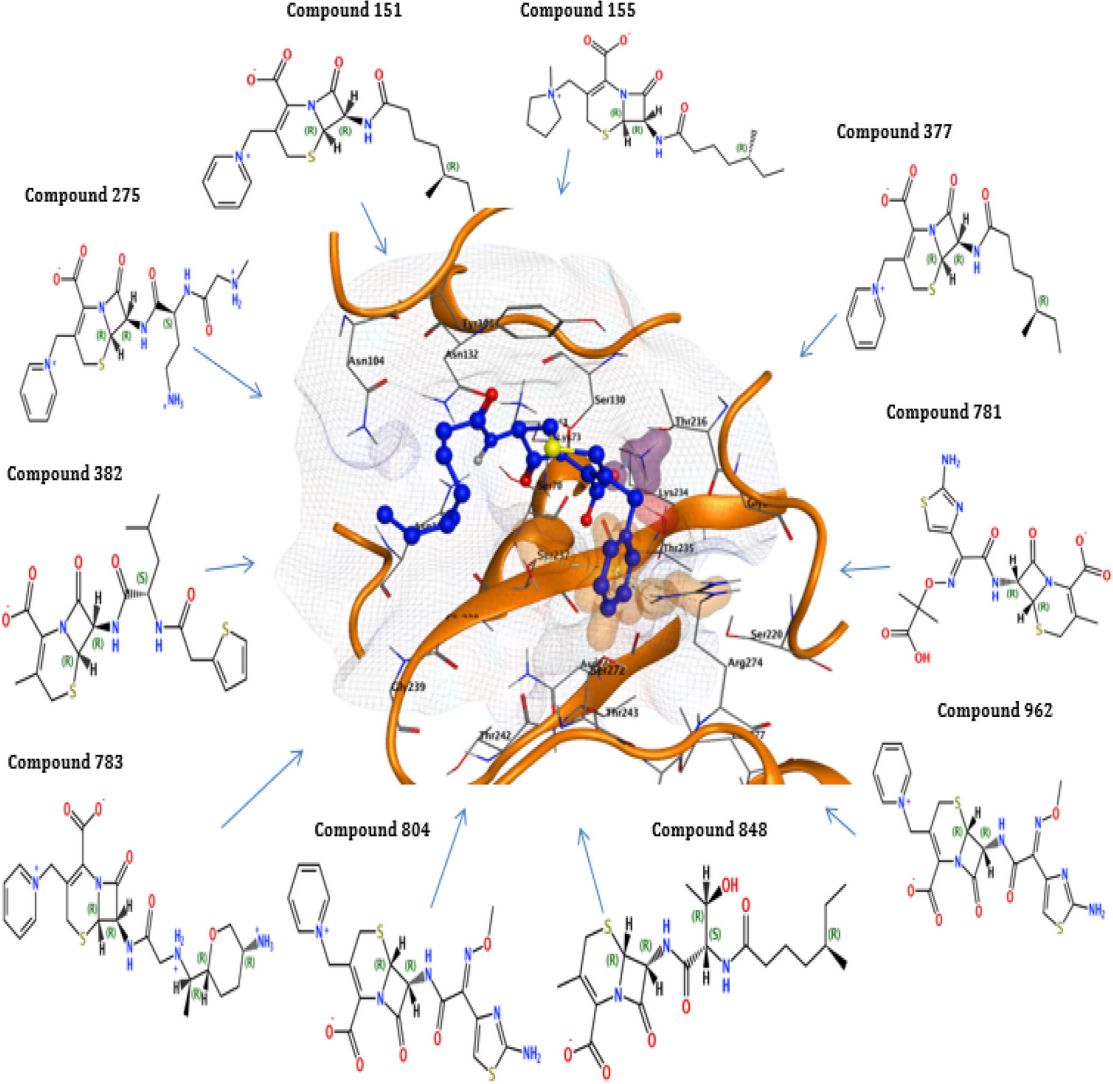
Interactions of the final retrieved hit compounds with target protein of Bla-CTX-M-15 after docking. The final ten compounds were predicted as promising hits compounds with the best ADMET properties, binding energy, and binding affinity, showing best interactions with Bla-CTX-M-15 protein. The final prominent hits compounds blocked the resistance mechanism of Bla-CTX-M-15 protein.

For the protein CTX-M-15, compound 783 has a binding energy of (−77.88 kcal/mol) with a binding affinity (−9.79 kcal/mol), compound 804 has a binding energy of (−71.44 kcal/mol) with a binding affinity (−9.21 kcal/mol). In the −65 to −69 kcal/mol binding energy range, two compounds are reported. In the −60 to −65 binding energy range, three compounds are reported. In −55 to −60 binding energy, two compounds were reported, compound 382 has binding energy (−58.60 kcal/mol) with affinity (−7.69 kcal/mol), and compound 377 has binding energy (−56.61 kcal/mol) with affinity (−8.01 kcal/mol). In the below −55 binning energy range has 1 compound was reported, compound 781 showed binding energy of (−53.40 kcal/mol) with affinity (−8.21 kcal/mol). All the ten reported compounds showed the best binding energy and affinity (Table 5). The compound 783 for Bla-CTX-M-15 protein observed the highest docking score of −8.21, which interact with Asn104, Tyr105, Asn,132, Lys234, Ser237, Tyr235, Gly236, and Arg274 of the active pocket having high binding energy (−77.88Kcal/mol) and affinity (−9.79Kcl/mol). The 2nd compound 804 for Bla-CTX-M-15 also documented a high docking score (−7.75), binding energy (−71.44Kcal/mol), and affinity (−9.21Kcal/mol), which interact with Ser70, Ser237, Tyr235, Asn104, Asn132, and Lys234 of the active pocket. The compounds 783 and 804 showed good interaction with the crucial residues of the Bla-CTX-M-15 active site (Tables 5 and Fig. 7).

### 3.12 Docking score, binding energy, and binding affinity for SHV-1 protein

**Fig. 8:**
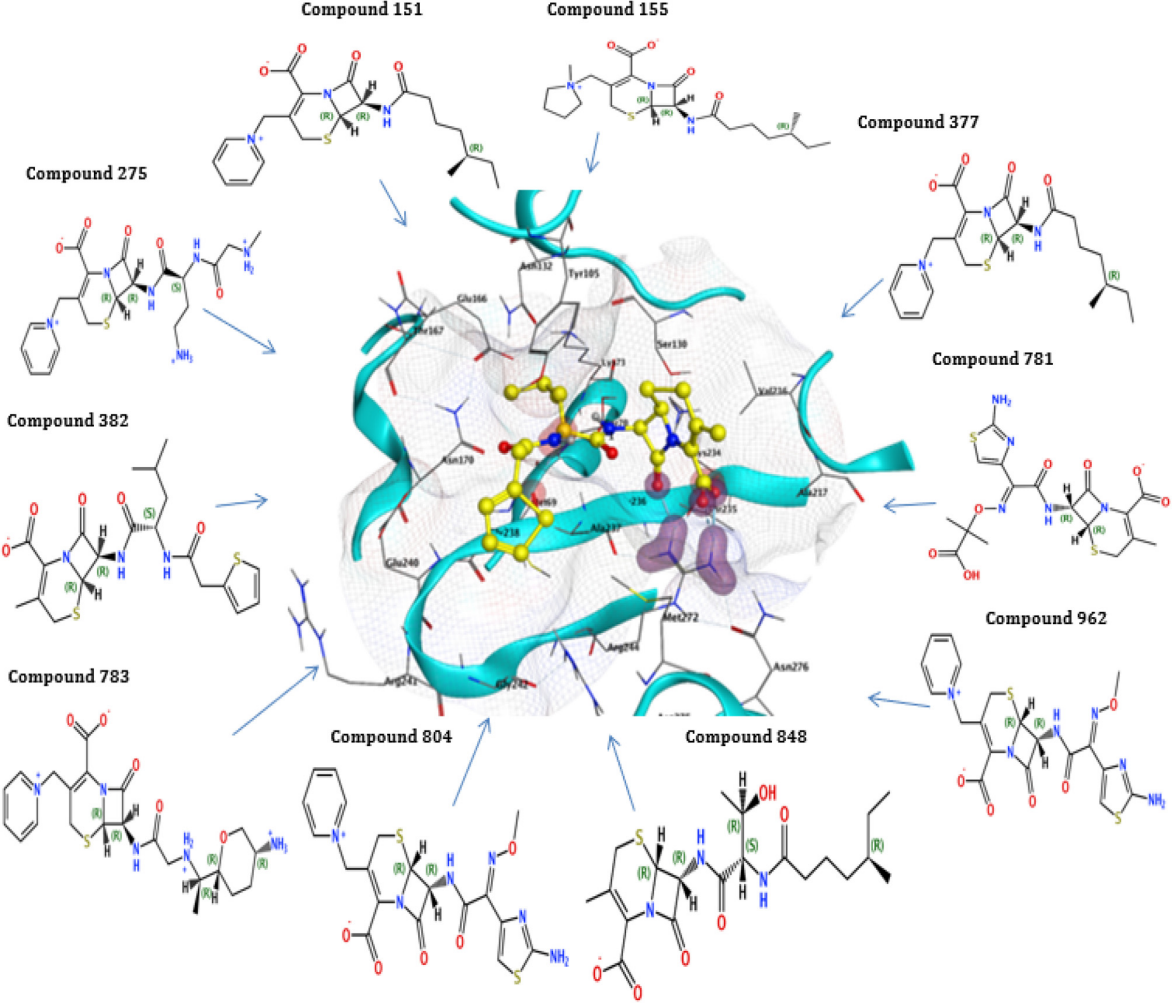
Interactions of the final retrieved hit compounds with target protein of Bla- SHV-1 after docking. The final ten compounds were predicted as promising hits compounds with the best ADMET properties, binding energy, and binding affinity, showing best interactions with Bla- SHV-1 protein. The final top hits compounds blocked the resistance mechanism of Bla- SHV-1 protein.

The selected compounds for bla-SHV-1, three compounds above −70 kcal/mol binding energy range were reported. Compound 275 have a binding energy of (−91.98 kcal/mol) with affinity (−11.03 kcal/mol), compound 783 have a binding energy of (−78.19 kcal/mol) with a binding affinity (−10.26 kcal/mol), and compound 804 have a binding energy of (−71.48 kcal/mol) with affinity (−9.47 kcal/mol). The reported ten compounds for the bla-SHV-1 protein showed the best interaction energies and stability (Table 5). The highest docking score (−7.45) of compound 848 was reported for Bla-SHV-1 with binding energy (−61.87Kcal/mol) and binding affinity (−8.47Kcal/mol) having interactions with Asn132, Glu166, Thr235, Ala237, Gly238, and Arg244 of the active pocket. The second compound 151 of Bla-SHV-1 presented docking score (−7.33), the binding energy of (−61.29Kcal/mol) and affinity (−8.37Kcl/mol) having interaction with Ser70, Lys234, Ala237, Met272 Asn132, and Arg244 of active pocket. Compounds 848 and 151 presented interaction with critical residues of the Bla-SHV-1 active site. Moreover, all the final hits also showed the best interactions with key residues of the active site of proteins (Tables 5 and Fig. 8).

## 4 Discussion

The frequency of ESBLs is increasing rapidly, develops an alarming condition because the ESBLs-Ec is challenging to inhibit due to resistance to antibiotics, cephalosporins, penicillins, and monobactam (Aboderin et al., 2009). The current study observed ESBLs production in 42.9% of *E. coli* isolates. The same results (35.8%) of ESBLs-Ec isolates were reported in a study conducted in Saudi Arabia (Hassan and Abdalhamid, 2014). Another study also noted the high prevalence of ESBL-Ec (36%) among clinical isolates in Pakistan, which aligns with our results (Pitout and Laupland, 2008). Another study conducted at Agha Khan University observed the exact prevalence (30%) of ESBLs-Ec isolates, according to the current research (Jabeen et al., 2005). Out of 246 isolates, 39.0% of patients were males, while 61% were females. Most of the ESBLs positive isolates were detected in hospitalized patients (53.2%) as compared to OPD patients (46.7%). The prevalence rates of ESBLs-Ec are different in different geographic areas among clinical isolates starting from low rates 3–8% in Sweden, Japan and Singapore to much higher rates 30–60% in America, 34% in Portugal, and 58% in Turkey (Paterson and Bonomo, 2005). Another study conducted in Pakistan also reported the high incidence (64%) of ESBLs-Ec isolates, supporting the current results. A much higher prevalence of ESBL detection (89.5%) was reported in Pakistan among clinically isolated *E. coli*. The results showed that most of the isolates were recovered from UTIs (61.4%) patients, followed by wound infected patients (pus) (29.3%), wound (1.6%), others (4.1%), upper and lower respiratory tract infections (3.3%) and chronic patients those developed ascites (1.22%). The results of the different clinical isolates of ESBLs-Ec revealed that 61.4% of patients had UTIs, 35.3% patients had SSIs, while 3.3% patients had severe pulmonary infections (PIs). However, these findings were in accordance with some other studies where the identical clinical isolates were recovered different specimens like; 46% from urine, 29% from pus, 11% from various fluids, and 4% from blood; while 12 isolates were obtained from throat swabs, catheter tips and sputum (Hussain et al., 2011, Mumtaz et al., 2011).

The antibiogram of the current study revealed that ESBLs-Ec isolates were 100% sensitive against TGC and MEM antibiotics, while high resistance was observed against FEP, CTX, ATM, CAZ, SXT, CIP, LEV, and AMC. The same findings were observed in which all the *E. coli* isolates were susceptible to IPM and MEM. The high resistance (89%) was recorded against CTX followed by CIP and FEP, which strongly supported the current study (Hussain et al., 2011). The present study observed MICs of antimicrobial agents tested against ESBLs-Ec isolates, which showed resistance to *β*-lactam and non-*β*-lactam drugs. Lower MICs were also reported in Athens (Greece), in which the majority of the strains also showed resistance to *β*-lactam and non-*β*-lactam drugs, while for TGC, MIC_50_ and MIC_90_ were 2 and 4 mg/ml, respectively (Galani et al., 2012). The same findings were also observed in a study conducted in South India in which a high resistance profile of the isolates was obtained; the MIC_50_ was high for cephalosporins and ATM, while MIC_50_ was low for TZP, FOS, IPM, MEM, and AK (8/4, 1, 0.5, 0.12, 0.06 and 2 mg/ml), respectively (Rajenderan et al., 2014).

The CTX-M gene was observed in 69.9%, TEM in 63.4%, SHV in 34.5% while CTX-M-14 in 17.5% isolates. The high incidence of 57.7% was reported in CTX-M, 20.3% in TEM and 15.4% in SHV in another reported study, making it in line with current findings (Sastry et al., 2013). The results of ESBL positive isolates showed a high rise in CTX-M (97.4%) compared to SHV (23.1%), which supports the current study (Hassan and Abdalhamid, 2014, Bindayna et al., 2010). It was reported that 44.08% of the isolates harbored the CTX-M genes alone, while both CTX-M and SHV genes were detected in 20.43% isolates. The increased incidence of the *E. coli* (59.32%) isolates harbored the CTX-M genes, which support the current study (Kaur and Aggarwal, 2013). It was observed that comparatively, a higher level of ESBL-producers was reported in the current study. Other different studies revealed the prominently different prevalence of ESBLs resistance genes in various geographical areas. This might be due to differences in drug selection and volume of ingestion of antibiotics and alterations in time of isolates collection.

In insilico drug drugs designing, the resistance to antibiotics is an issue of concern and has involved interests for researchers worldwide. In the past, insilico approaches have been used to predict strategies like targeting the protein crystal structures to design novel inhibitors to overcome the mechanism of resistance (Hassan Baig et al., 2016). The results of new compounds as inhibitors revealed that the reported ligand for the bla-TEM Ethyl Boronic Acid has binding energy (−56.72Kcal/mol) and affinity (−8.43 kcal/mol). The highest binding energy (−80.49 kcal/mol) and affinity (−9.48 kcal/mol) were reported for bla-TEM of compound 275, followed by compound having 783 binding energy (−78.66 kcal/mol), affinity (−9.43 kcal/mol), compound 848 with binding energy (−70–84 kcal/mol), affinity (−8.98 kcal/mol). These three compounds showed binding energy above −70 kcal/mol. All the ten reported compounds showed the best binding energy and affinity compared to the reported ligand Ethyl Boronic Acid. In the docking simulations, the final docking hits observed the best interactions with each protein (essential amino acids). The docking result of compound 848 for TEM protein revealed the highest docking score of −8.08, high binding energy (−70.84Kcal/mol), and affinity (−8.98Kcl/mol). The compound 783 for Bla-TEM also documented a high docking score (−8.03), binding energy (−78.66Kcal/mol), and affinity (−9.43Kcal/mol). The compounds 848 and 783 recorded the best interaction with crucial residues of TEM active site than the reported ligand Ethyl Boronic Acid (Table 5).

The reported ligand ruthenium showed binding energy of (−50.69 kcal/mol) with affinity (−8.37 kcal/mol) for the protein bla-CTX-M-14. The results of 4 compounds out of 10 selected compounds for bla-CTX-M-14 revealed that compound 962 has binding energy (−73.81 kcal/mol) with affinity (−9.67 kcal/mol), the compound 848 has a binding energy of (−72.54 kcal/mol) with affinity (−9.73 kcal/mol), the compound 275 have a binding energy of (−71.42 kcal/mol) with affinity (−8.55 kcal/mol) and compound 781 have a binding energy of (−70.74 kcal/mol) with affinity (−9.02 kcal/mol) have binding energy above that −70 kcal/mol. All the ten reported compounds showed the best binding energy and affinity compared to the reported ligand ruthenium. The highest docking score (−7.30) of compound 783 was observed for Bla-CTX-M-14 with binding energy (−69.95Kcal/mol) and affinity (−8.63Kcal/mol). The second compound, 962 of Bla-CTX-M-14, documented docking score (−7.28), binding energy (−73.81Kcal/mol), and affinity (−9.67Kcl/mol). The compounds 783 and 962 showed good interaction with the critical residues of Bla- CTX-M-14 active site than the reported ligand ruthenium (Table 5).

The reported ligand of the bla-CTX-M-15 (2S, 5R)-1-formyl-5-[(sulfooxy)amino] piperidine-2-carboxamide (NXL) have a binding energy of (−46.89 kcal/mol) with an affinity of (−6.63 kcal/mol). The ten selected compounds showed that binding energy and affinity are more significant than the reported ligand NXL. The compound 783 for Bla-CTX-M-15 protein observed the highest docking score of −8.21, high binding energy (−77.88Kcal/mol), and affinity (−9.79Kcl/mol). The 2nd compound 804 for Bla-CTX-M-15 also documented a high docking score (−7.75), binding energy (−71.44Kcal/mol), and affinity (−9.21Kcal/mol). The compounds 783 and 804 showed good interaction with the essential residues of the Bla-CTX-M-15 active site than the reported ligand NXL (Table 5).

The reported ligand of the bla-SHV-1 Meropenem has a binding energy of (−50.05 kcal/mol) with a binding affinity (−6.63 kcal/mol). Comparing the reported ligand with the selected compounds for bla-SHV-1, three compounds above −70 kcal/mol binding energy range were reported. Compound 275 have a binding energy of (−91.98 kcal/mol) with affinity (−11.03 kcal/mol), compound 783 have a binding energy of (−78.19 kcal/mol) with a binding affinity (−10.26 kcal/mol), and compound 804 have a binding energy of (−71.48 kcal/mol) with affinity (−9.47 kcal/mol). The reported ten compounds for the bla-SHV-1 protein showed the best interaction energies and stability compared to the reported ligand Meropenem of bla-SHV-1. The highest docking score (−7.45) of compound 848 was reported for Bla-SHV-1 with binding energy (−61.87Kcal/mol) and binding affinity (−8.47Kcal/mol). The second compound 151 of Bla-SHV-1 presented a docking score (−7.33), the binding energy of (−61.29Kcal/mol), and affinity (−8.37Kcl/mol). Compounds 848 and 151 presented interaction with crucial residues of the Bla-SHV-1 active site compared to the reported ligand Meropenem (Table 5).

## 5 Conclusions

The ESBLs-Ec is an issue of concern for clinicians to treat resistant bacteria due to misuse and self-medications. Due to the emergence of resistance mechanisms, it is challenging for medical practitioners to treat patients with a limited choice of antibiotics. The researchers designed many new drugs for ESBLs-Ec to overcome the resistance mechanism. The proteins of ESBLs are the cause of different infections in both hospitalized and non-hospitalized patients. Thus it is necessary to overcome the resistance mechanism to treat the patients. In the current study, the in-silico approach was used to discover novel and potent inhibitors for TEM, CTX-M-14, CTX-M-15, and SHV-1 proteins. The study results revealed that 40 compounds were designed as final hits (10 for each protein). These hits compounds have unique scaffolds and are predicted to be starting points for developing novel and potent inhibitors for four antibiotic-resistant proteins.

## 6 Ethics approval

The current study was approved by the Institution Research and Ethical Review Board (IREB) of Khyber Medical College, Peshawar (Document No. 122/ADR/KMC).

## 7 Consent to participate

All authors consent to participate in this manuscript.

## 8 Consent for publication

All authors consent to publish this manuscript in the Saudi Journal of Biological sciences.

## 9 Availability of data and material

Data will be available on request to the corresponding author or first author.

## 10 Code availability

Not applicable.

## Declaration of Competing Interest

The authors declare that they have no known competing financial interests or personal relationships that could have appeared to influence the work reported in this paper.
